# Supplementary material for: Altered microRNA Transcriptome in Cultured Human Liver Cells upon Infection with Ebola Virus
Source: Int J Mol Sci. 2021 Apr 6;22(7):3792. doi: 10.3390/ijms22073792 (PMC8038836; doi:10.3390/ijms22073792)
Supplement: Supplementary file 1 [file ijms-22-03792-s001.zip › Supplementary_File/D_ KEGG_Analysis_Results/16-30nt_pathway_Makona-24h-Huh7_vs_Control-24h-Huh7_down.mature_mirna_targets/hsa_pathwayResult.html]

| PathwayID | Definition | OriginalWebSite | Fisher-Pvalue | SelectionCounts | SelectionSize | Count | Size | FDR | Enrichment\_Score | GeneRatio | Genes |
| --- | --- | --- | --- | --- | --- | --- | --- | --- | --- | --- | --- |
| hsa04666 | Fc gamma R-mediated phagocytosis - Homo sapiens (human) | http://www.genome.jp/kegg-bin/show\_pathway?hsa04666+10094+55616+10451 | 1.660942e-02 | 3 | 46 | 93 | 7906 | 1.000000e+00 | 1.779646 | 0.065217 | ARPC3//ASAP3//VAV3 |
| hsa04660 | T cell receptor signaling pathway - Homo sapiens (human) | http://www.genome.jp/kegg-bin/show\_pathway?hsa04660+3845+6885+10451 | 2.174509e-02 | 3 | 46 | 103 | 7906 | 1.000000e+00 | 1.662639 | 0.065217 | KRAS//MAP3K7//VAV3 |
| hsa03050 | Proteasome - Homo sapiens (human) | http://www.genome.jp/kegg-bin/show\_pathway?hsa03050+5696+5706 | 2.798112e-02 | 2 | 46 | 45 | 7906 | 1.000000e+00 | 1.553135 | 0.043478 | PSMB8//PSMC6 |
| hsa04722 | Neurotrophin signaling pathway - Homo sapiens (human) | http://www.genome.jp/kegg-bin/show\_pathway?hsa04722+3845+5598+11108 | 3.157848e-02 | 3 | 46 | 119 | 7906 | 1.000000e+00 | 1.500609 | 0.065217 | KRAS//MAPK7//PRDM4 |
| hsa04152 | AMPK signaling pathway - Homo sapiens (human) | http://www.genome.jp/kegg-bin/show\_pathway?hsa04152+8789+6885+5862 | 3.225898e-02 | 3 | 46 | 120 | 7906 | 1.000000e+00 | 1.491349 | 0.065217 | FBP2//MAP3K7//RAB2A |
| hsa00270 | Cysteine and methionine metabolism - Homo sapiens (human) | http://www.genome.jp/kegg-bin/show\_pathway?hsa00270+10768+4507 | 3.275581e-02 | 2 | 46 | 49 | 7906 | 1.000000e+00 | 1.484712 | 0.043478 | AHCYL1//MTAP |
| hsa04979 | Cholesterol metabolism - Homo sapiens (human) | http://www.genome.jp/kegg-bin/show\_pathway?hsa04979+9218+9217 | 3.399628e-02 | 2 | 46 | 50 | 7906 | 1.000000e+00 | 1.468569 | 0.043478 | VAPA//VAPB |
| hsa05110 | Vibrio cholerae infection - Homo sapiens (human) | http://www.genome.jp/kegg-bin/show\_pathway?hsa05110+523+10945 | 3.399628e-02 | 2 | 46 | 50 | 7906 | 1.000000e+00 | 1.468569 | 0.043478 | ATP6V1A//KDELR1 |
| hsa03460 | Fanconi anemia pathway - Homo sapiens (human) | http://www.genome.jp/kegg-bin/show\_pathway?hsa03460+5429+11201 | 3.913803e-02 | 2 | 46 | 54 | 7906 | 1.000000e+00 | 1.407401 | 0.043478 | POLH//POLI |
| hsa04730 | Long-term depression - Homo sapiens (human) | http://www.genome.jp/kegg-bin/show\_pathway?hsa04730+2892+3845 | 4.736394e-02 | 2 | 46 | 60 | 7906 | 1.000000e+00 | 1.324552 | 0.043478 | GRIA3//KRAS |
| hsa05416 | Viral myocarditis - Homo sapiens (human) | http://www.genome.jp/kegg-bin/show\_pathway?hsa05416+27+3113 | 4.736394e-02 | 2 | 46 | 60 | 7906 | 1.000000e+00 | 1.324552 | 0.043478 | ABL2//HLA-DPA1 |
